# Challenges in Diagnosing and Managing Acute Cholecystitis in Cirrhosis

**DOI:** 10.7759/cureus.80870

**Published:** 2025-03-20

**Authors:** Angsupat Pornchai, Nicha Wongjarupong, Adil S Mir, Evelyn J Calderon Martinez, John Cinicola

**Affiliations:** 1 Internal Medicine, University of Pittsburgh Medical Center Pinnacle, Harrisburg, USA; 2 Gastroenterology, Cedars-Sinai Medical Center, Los Angeles, USA; 3 Gastroenterology, Virginia Tech Carilion School of Medicine, Roanoke, USA

**Keywords:** acute calculus cholecystitis, endoscopic ultrasound-guided gallbladder drainage, hida cholescintigraphy, lap cholecystectomy, live cirrhosis

## Abstract

A 64-year-old male with cirrhosis and mild ascites secondary to metabolic-associated steatotic liver disease (MASLD) presented with right upper quadrant (RUQ) abdominal pain. Initial investigations, including an abdominal ultrasound and hepatobiliary iminodiacetic acid (HIDA) scan, did not show any evidence of acute cholecystitis. However, the patient’s persistent symptoms and a positive sonographic Murphy’s sign raised clinical suspicion for the condition. Subsequently, a computed tomography (CT) scan confirmed the diagnosis of acute cholecystitis. Blood cultures revealed *Enterococcus faecalis* and *Klebsiella pneumoniae*, prompting targeted antibiotic therapy. Given the high operative risk associated with cirrhosis, ascites, and extensive varices, the patient was treated conservatively with intravenous antibiotics followed by oral antibiotics. He was discharged with plans for an elective laparoscopic cholecystectomy versus endoscopic ultrasound-guided cholecystostomy (EUS-GBD) after further optimization for potential liver transplantation at an advanced center. This case underscores the complexities of diagnosing acute cholecystitis in cirrhotic patients, highlights the need for vigilant re-evaluation when imaging and clinical findings diverge, and addresses the challenges of managing high-risk surgical patients.

## Introduction

Acute cholecystitis is an inflammation of the gallbladder, typically resulting from cystic duct obstruction by a gallstone, leading to bile accumulation, increased pressure in the gallbladder, and compromised blood flow. This inflammatory response can cause right upper quadrant abdominal pain, fever, and leukocytosis, which may worsen if bacterial growth exacerbates the inflammation. Physical examination often reveals Murphy’s sign (pain and inspiratory arrest on right upper quadrant palpation). If left untreated, serious complications, such as gallbladder necrosis or perforation, can occur. It is often diagnosed by ultrasound and confirmed by a hepatobiliary iminodiacetic acid (HIDA) scan. Recent guidelines highlight ultrasound as the first-line imaging modality, especially for early or uncomplicated cases [1]. Although acute cholecystitis is not a direct cause of cirrhosis, it can contribute to liver issues through recurrent inflammation or prolonged biliary obstruction. Cirrhosis is the end-stage anatomical and functional disruption of the liver, characterized by progressive fibrosis, nodular regeneration, and resultant impairment of hepatic function. Chronic conditions like viral hepatitis, alcohol use, or metabolic-associated steatotic liver disease (MASLD) result in persistent inflammation, scar formation, and accumulation of collagen in the parenchyma, transforming normal hepatic architecture into a distorted, nodular structure that predisposes individuals to complications like portal hypertension and, ultimately, liver failure. In patients with compromised hepatic function, however, the accuracy of these diagnostic modalities can be significantly reduced due to altered tracer excretion and possible imaging artifacts [2]. Studies indicate that cirrhosis, particularly in its advanced stages, can amplify such limitations, leading to false-positives or false-negative findings that mimic obstruction or mask acute inflammation [3]. We present a case that illustrates how cirrhosis can confound imaging findings of acute cholecystitis, underscores the necessity of maintaining a high index of suspicion when imaging is inconclusive, and exemplifies the critical role of clinical judgment in balancing the risks of surgery with conservative treatment [4].

## Case presentation

A 64-year-old male with cirrhosis secondary to MASLD presented to an emergency department with recurrent right upper quadrant abdominal pain, na­­usea, and vomiting for the past eight hours. His cirrhosis is complicated by portal hypertension, evidenced by splenomegaly, trace ascites, extensive splenorenal varices, and a history of a failed transjugular intrahepatic portosystemic shunt (TIPS) complicated by an injured splenic artery requiring splenic artery embolization in June 2022 prior to the presentation. His model for end-stage liver disease (MELD) 3.0 score was 18 with a Child-Pugh score of 9 (class B).

He also has mild gastric antral vascular ectasia, which was treated with argon plasma coagulation, as well as atrial fibrillation, for which he is not on anticoagulation due to a recent history of variceal bleeding, along with chronic type 2 diabetes mellitus, and chronic hypertension. He was under evaluation for liver transplantation regarding decompensated cirrhosis with variceal bleeding at an advanced care center at the time of presentation. He additionally has a past medical history of biliary colic, with cholelithiasis noted on a prior abdominal ultrasound.

On admission, he was fully alert and oriented, afebrile, and hemodynamically stable. On examination, the abdomen was soft, with marked tenderness over the right upper quadrant area, no rebound tenderness, and normal bowel sounds. Laboratory results showed a white blood cell count of 6.5 × 10^3/μL, hemoglobin of 12.1 g/dL, platelet count of 71 K/μL, international normalized ratio (INR) of 1.6, blood urea nitrogen (BUN) of 26 mg/dL, creatinine of 1.14 mg/dL at baseline, alkaline phosphatase of 128 U/L, alanine aminotransferase (ALT) of 15 U/L, aspartate aminotransferase (AST) 40 U/L, total bilirubin of 2.8 mg/dL, direct bilirubin of 1.1 mg/dL, albumin of 2.1 g/dL, ammonia of 96 μmol/L, and lipase of 34 U/L (Table 1).

**Table 1:** Hematology and blood chemistry report INR: international normalized ratio, BUN: blood urea nitrogen, ALP: alkaline phosphatase, ALT: alanine aminotransferase, AST: aspartate aminotransferase

Test	Result	Reference Range	Units
White Blood Cell Count	6.5 × 10^3/μL	3.9-9.5	K/μL
Hemoglobin	12.1	12.8-16.6	g/dL
Platelet Count	71	140-366	K/μL
INR	1.6	0.8-1.1	
BUN	26	7-25	mg/dL
Creatinine	1.14	0.7-1.3	mg/dL
Alkaline Phosphatase	128	23-127	U/L
ALT	15	7-52	U/L
AST	40	13-39	U/L
Total Bilirubin	2.8	0.3-1.0	mg/dL
Direct Bilirubin	1.1	0-0.2	mg/dL
Albumin	2.1	3.5-5.7	g/dL
Ammonia	96	<47	μmol/L
Lipase	34	11-82	U/L

**Figure 1:**
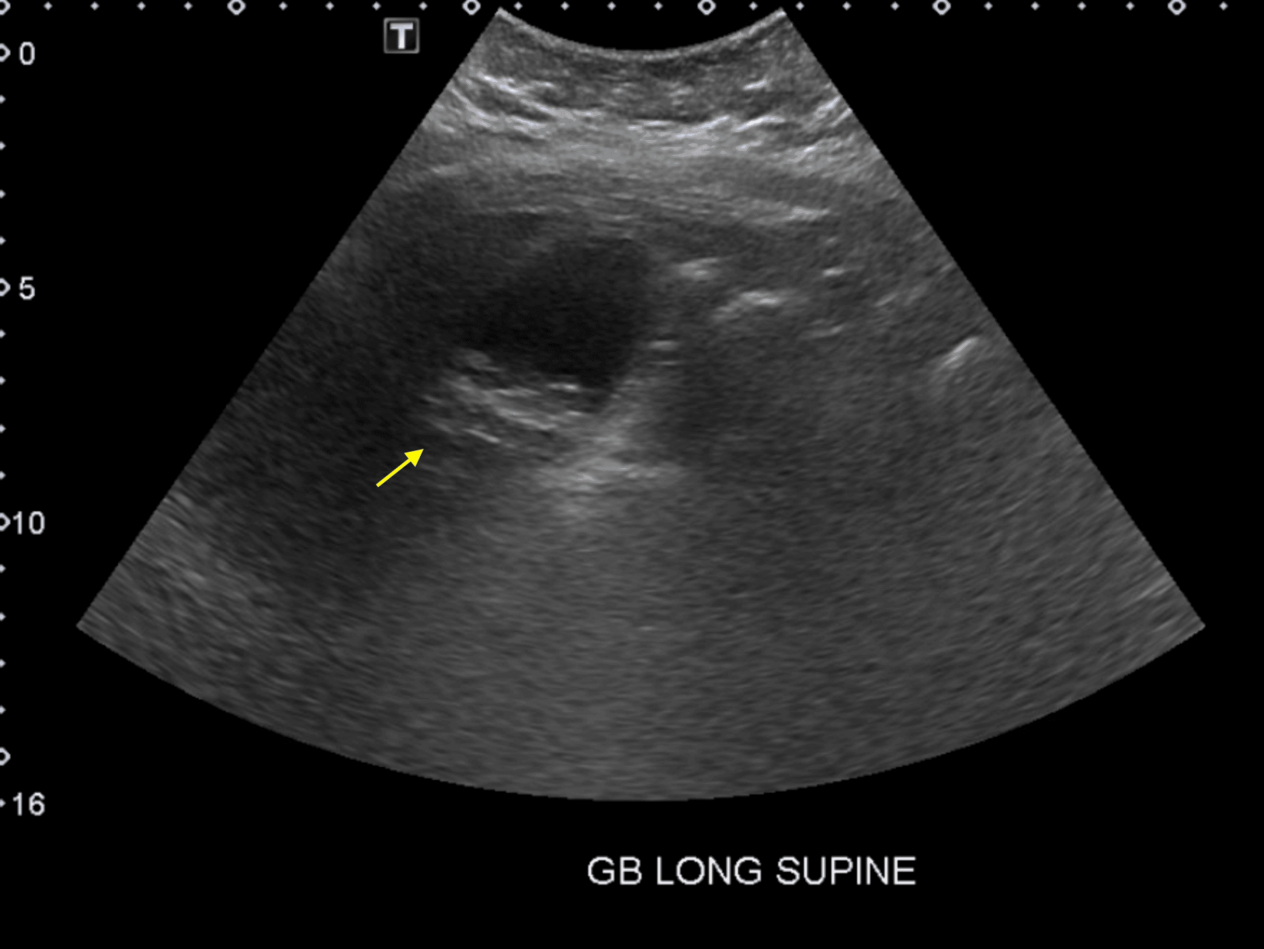
Gallbladder ultrasound in a long supine position Multiple echogenic, shadowing gallstones within gallbladder lumen; no pericholecystic fluid or gallbladder wall thickening

**Figure 2:**
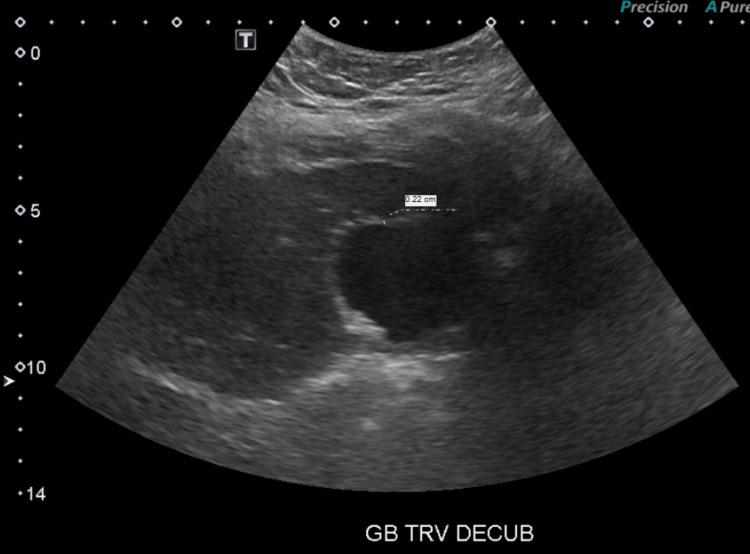
Gallbladder ultrasound in a transverse decubitus position The gallbladder wall measures 2 mm, and there is no pericholecystic fluid.

**Figure 3:**
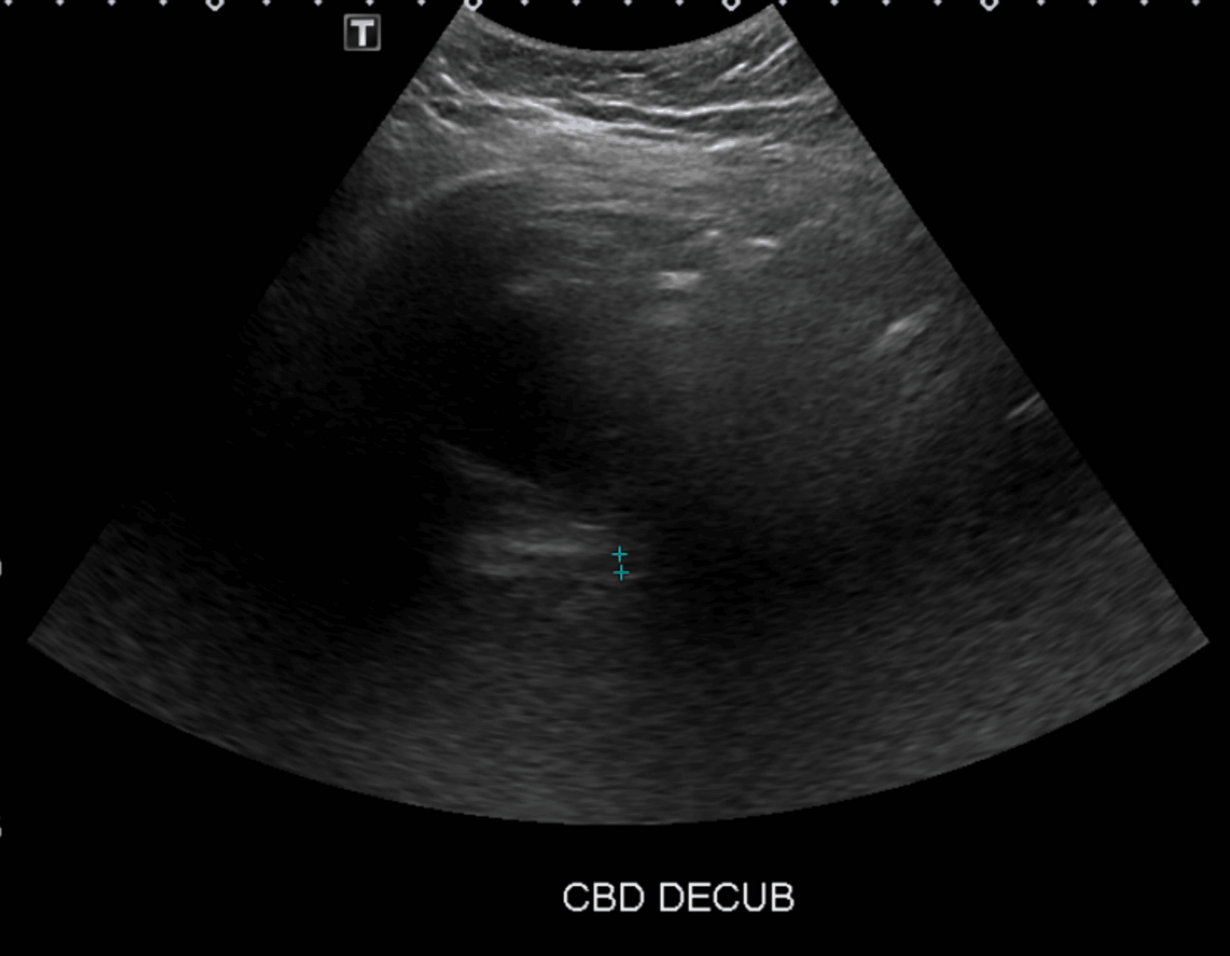
Gallbladder ultrasound in a decubitus position CBD measures 4 mm, which is within normal limits.

**Figure 4:**
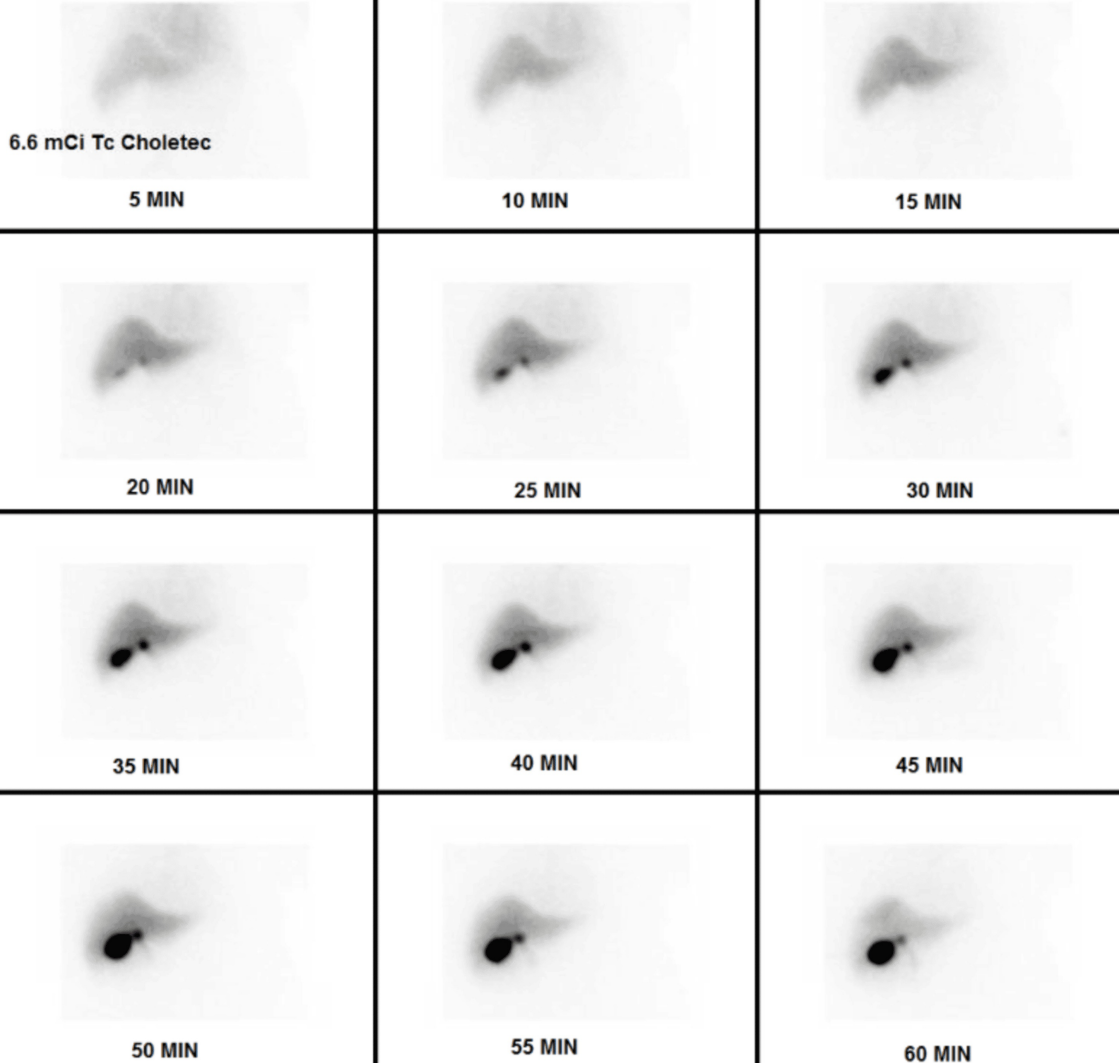
HIDA (hepatobiliary iminodiacetic acid) scan There is no scintigraphic evidence of acute cholecystitis. There is prompt and homogeneous uptake of activity in the liver. The gallbladder is visualized at 60 minutes. No definite small bowel activity is seen at 60 minutes.

**Figure 5:**
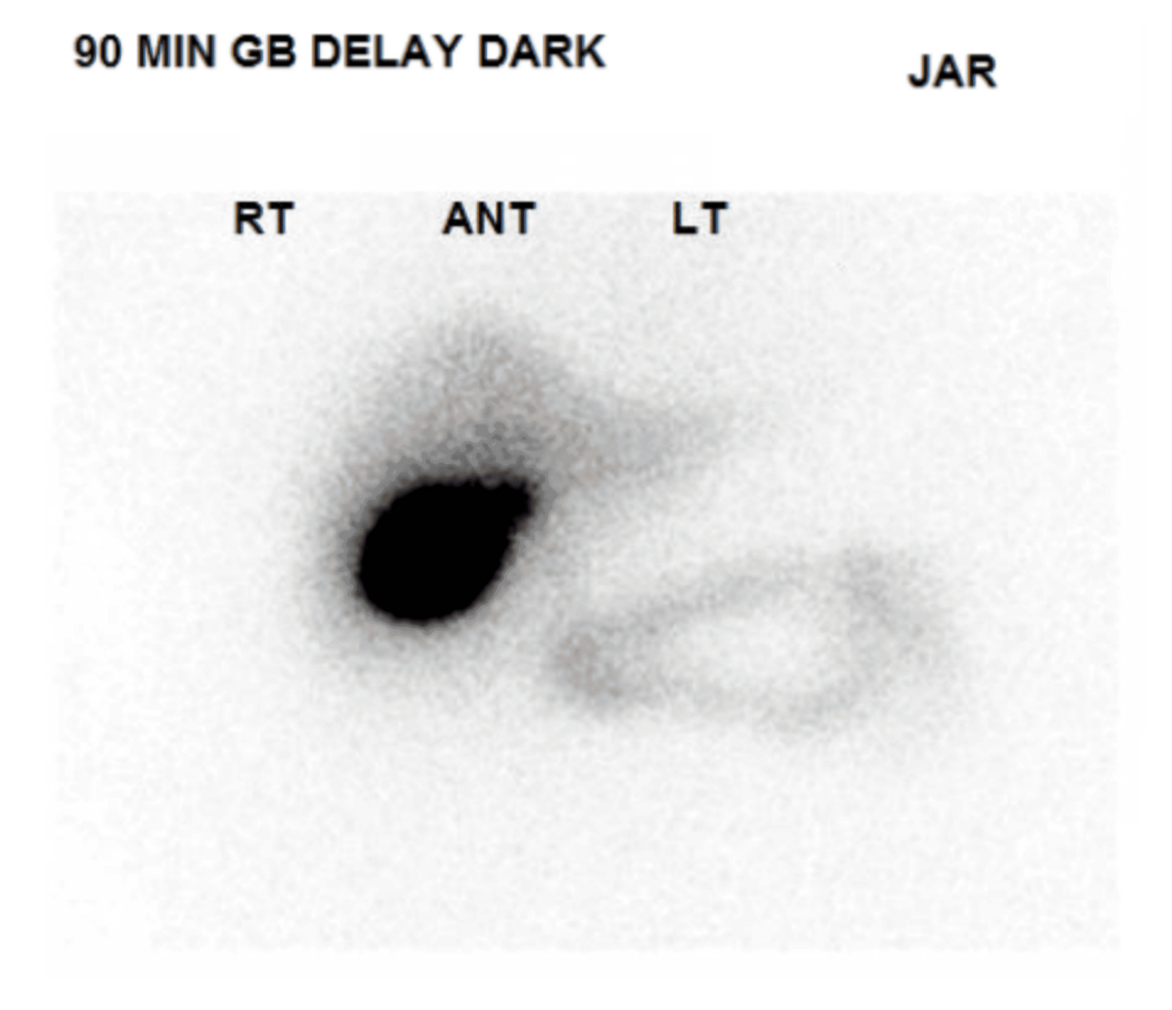
HIDA (hepatobiliary iminodiacetic acid) scan There is some radiotracer in the upper abdomen, likely within the small bowel, at 90 minutes.

**Figure 6:**
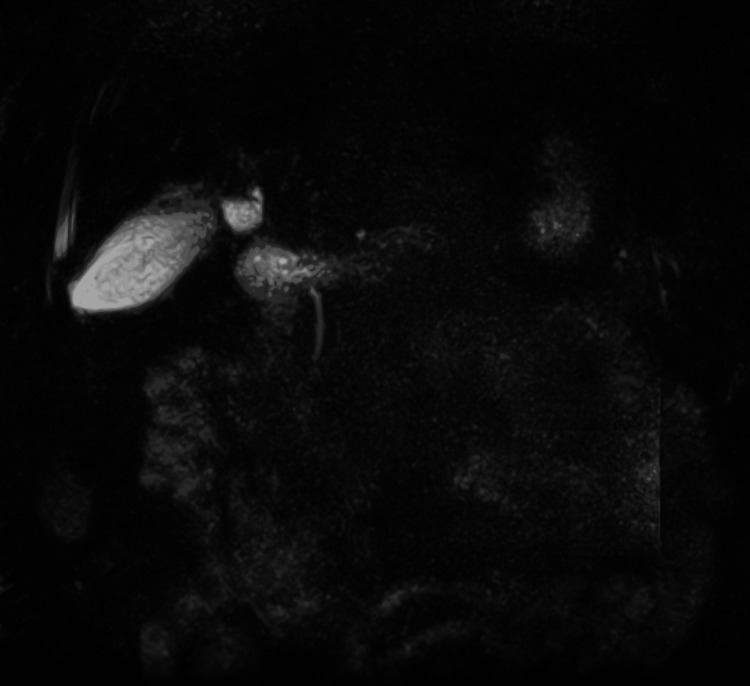
Magnetic resonance cholangiopancreatography There is no biliary duct obstruction or cholelithiasis.

Abdominal ultrasound showed cholelithiasis (Figure 1) with no gallbladder wall thickening or pericholecystic fluid (Figure 2) and no dilatation of the biliary ducts (Figure 3), but a positive sonographic Murphy’s sign indicated possible acute cholecystitis. A HIDA scan showed homogeneous uptake of radiotracer by the liver, with visualization of the gallbladder at 60 minutes. No definitive small bowel activity was observed at 60 minutes (Figure 4); however, delayed images showed radiotracer in the upper abdomen, likely within the small bowel (Figure 5). There was no scintigraphic evidence of acute cholecystitis but delayed small bowel activity was suspected and partial biliary obstruction or stricture could not be entirely excluded. MRCP revealed small gallstones, with no evidence of cholecystitis, biliary ductal dilation, or choledocholithiasis (Figure 6). Despite these findings, the patient continued to have persistent abdominal pain and a positive Murphy’s sign. Because the patient was afebrile and hemodynamically stable, the surgical team recommended clinical observation without antibiotics, a diet challenge, and potential discharge the following day.

**Figure 7:**
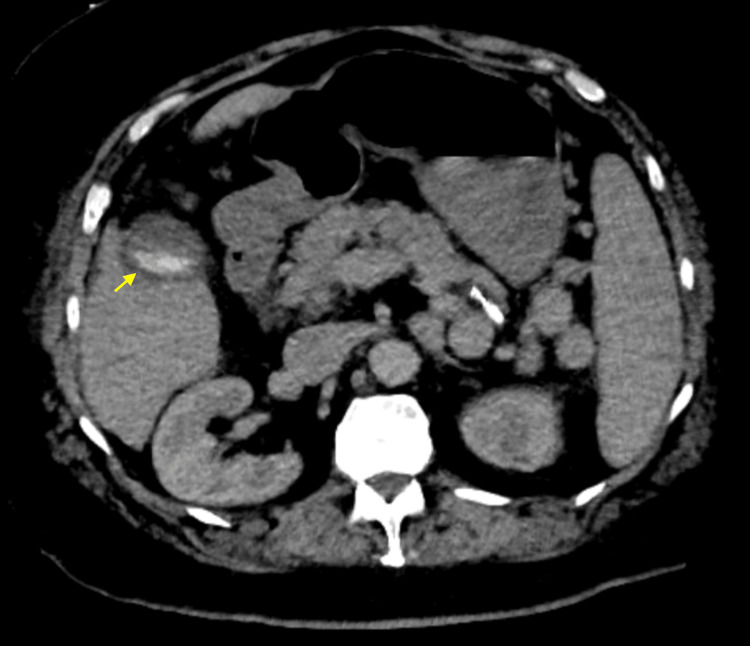
Axial contrast-enhanced CT abdomen Gallstones are present, along with some thickening and edema of the gallbladder wall. There is no biliary duct dilation or choledocholithiasis.

However, that evening, the patient developed fever, chills, shortness of breath, and palpitations, with a temperature of 38.3 °C, BP of 160/70 mmHg, HR of 180 bpm, tachypnea (RR 24/min), and hypoxia in the high 80s, requiring 2 L of oxygen via a nasal cannula. An electrocardiogram revealed atrial fibrillation with rapid ventricular response. Acetaminophen 650 mg was administered at the time. IV metoprolol 5 mg was given twice, resulting in mild improvement of the heart rate to the 140s, and a diltiazem drip was started. Cardiology was also consulted. A repeat computed tomography (CT) scan showed gallbladder wall thickening and edema consistent with acute cholecystitis, but no choledocholithiasis or bile duct dilation (Figure 7). Empiric intravenous antibiotics (ceftriaxone 2 grams daily and metronidazole 500 milligrams twice daily) were initiated, and subsequent blood cultures grew *Enterococcus faecalis* and *Klebsiella pneumoniae*. The patient was switched to oral amoxicillin-clavulanate (875 mg/125 mg, Augmentin) every 8 hours after a 4-day course of intravenous antibiotics, as the patient remained afebrile, showed resolution of leukocytosis (WBC of 4 K/μL), maintained hemodynamic stability during antibiotic treatment, and antibiotic susceptibility was confirmed. A repeat blood culture obtained 48 hours later demonstrated no growth. The patient also received Tylenol 650 mg every 6 hours as needed, morphine 2 mg as needed for pain control, and ondansetron 4 mg as needed for nausea and vomiting.

The patient’s combination of cirrhosis from MASLD requiring transplantation evaluation, recent variceal bleeding, and multiple comorbidities renders him a high-risk surgical candidate for cholecystectomy. Due to clinical improvement, gallbladder drainage intervention was deferred during the index admission. The patient received a total of nine days of antibiotics during the hospital stay and was discharged on oral Augmentin to complete a two-week course of antibiotics with plans for an outpatient laparoscopic cholecystectomy or endoscopic ultrasound-guided cholecystostomy (EUS-GBD) at an advanced care center, where he is evaluated for liver transplantation.

## Discussion

Diagnosing acute cholecystitis in patients with cirrhosis poses unique challenges, primarily because of altered hepatic function and the secondary changes associated with portal hypertension [1]. In cirrhotic patients, particularly those with MASLD, the diagnosis of acute cholecystitis can be complex due to altered hemodynamics, impaired immune responses, and shifts in hepatic architecture that dull normal inflammatory markers or mask typical imaging findings. The literature consistently shows that gallbladder disease in this population often presents atypically, delaying diagnosis and contributing to higher morbidity. This patient’s persistent and localized right upper quadrant (RUQ) abdominal pain, coupled with a positive sonographic Murphy’s sign despite inconclusive ultrasound, HIDA, and MRCP results, underscores the importance of maintaining a high degree of clinical suspicion for evolving cholecystitis in cirrhosis. Additionally, differential diagnoses ranging from ascending cholangitis to hepatic abscess must be thoroughly considered, given their similar presentation and the fact that cirrhosis may simultaneously heighten vulnerability to biliary infections [3,5,6]. Ultimately, the patient’s clinical deterioration and repeat imaging demonstrated acute cholecystitis, underscoring the limitations of conventional imaging modalities in cirrhotic patients, and highlighting the importance of continued clinical vigilance [7].

In individuals with impaired hepatic function, both ultrasound and HIDA scans can yield false-negative or false-positive results. This issue arises from altered tracer extraction and excretion by a diseased liver. In such circumstances, liver cells are significantly compromised, leading to reduced uptake of the radioactive tracer and diminished excretion into the biliary system. These functional changes can cause a clinically confusing pattern on HIDA scanning. In many cases, the gallbladder may not fill on standard imaging, creating a picture that resembles cystic duct obstruction and mimics acute cholecystitis (i.e., a false-positive). By contrast, erratic or limited tracer distribution might mask an actual pathology, such as inflammation or partial obstruction, generating a false-negative picture. Consequently, clinicians should be cautious when interpreting HIDA scans in patients with advanced chronic liver disease [2,3,8-10]. Similarly, subtle gallbladder wall changes may not be readily apparent on ultrasound when cirrhosis-related changes, such as ascites, varices, and altered gallbladder anatomy, coexist [1]. Therefore, clinicians should interpret imaging results with caution in patients who have advanced liver disease and maintain a high index of suspicion for acute cholecystitis, especially when clinical findings (e.g., positive sonographic Murphy’s sign, persistently severe RUQ abdominal pain) and laboratory parameters (rising inflammatory markers or signs of sepsis) point to active inflammation [11].

An essential lesson from this case is the need for interval clinical reassessment and, if needed, a repeat or additional cross-sectional imaging when there is a mismatch between clinical presentation and initial imaging results [5,12]. By maintaining a low threshold for further or repeated studies, new or evolving pathologies may be detected before they manifest as significant complications such as sepsis. In this patient, a repeat CT scan was pivotal in identifying gallbladder wall thickening and edema, thereby confirming acute cholecystitis when earlier studies were inconclusive [7].

Management decisions in patients with cirrhosis who have acute surgical conditions also require careful balancing of surgical risks and the risks of nonoperative management [11]. This patient’s high MELD 3.0 score, cirrhosis, evidence of portal hypertension, extensive varices, and multiple comorbidities made him a poor candidate for cholecystectomy. In the context of high-risk surgical patients with acute cholecystitis, endoscopic ultrasound-guided gallbladder drainage (EUS-GBD) has gained prominence as a less invasive but equally effective alternative to percutaneous transhepatic gallbladder drainage (PT-GBD). By creating a controlled fistula via lumen-apposing metal stents, EUS-GBD significantly minimizes reliance on external drains and associated complications such as leakage, skin irritation, and patient discomfort. It also mitigates the risk of recurrent cholecystitis often seen in PT-GBD when catheters dislodge or occlude. Furthermore, with EUS-GBD ensuring precise targeting of the gallbladder and secure stent placement, the likelihood of procedure-related complications is comparatively low, and the patient’s recovery is often more straightforward. Notable complications can arise both early and late. Early adverse events include bleeding, bile leakage with potential peritonitis, infection, pneumoperitoneum, and perforation. Late complications primarily involve stent-related issues: migration, occlusion, and recurrent cholecystitis. While EUS-GBD may initially appear technically challenging, particularly in cirrhotic patients or those with large ascites, the procedure has demonstrated reproducible outcomes in centers with advanced endoscopy expertise. As such, it stands out as a feasible, efficacious, and patient-friendly approach for gallbladder decompression in those who cannot tolerate cholecystectomy [13,14].

Finally, treatment decisions must draw upon a multidisciplinary framework in which surgery, hepatology, interventional radiology, and gastroenterology collaborate to tailor interventions optimally [15]. In this patient, conservative management with antibiotics and a delayed outpatient cholecystectomy or cholecystostomy once feasible was selected after addressing decompensating factors, reflecting careful risk-benefit considerations. This conservative approach, guided by a multidisciplinary team including general surgery, gastroenterology, and infectious diseases, highlights the importance of individualized treatment strategies for high-risk patients [1,12].

Although this case offers important insights into the complex interplay between cirrhosis and gallbladder pathology, it is primarily limited by the single-patient focus, which may affect generalizability to broader patient populations. Imaging challenges stemming from compromised hepatic function, portal hypertension, and altered anatomy underscore the need for caution and further validation in larger cohorts. Additionally, expertise biases may exist; for instance, the technical success of EUS-GBD can vary across centers, limiting immediate adoption where advanced endoscopic proficiency is lacking.

Future suggestions involve the increased use of EUS-GBD as a less invasive yet effective option for gallbladder decompression, particularly in high-risk patients who are poor candidates for surgery. The recommended protocol emphasizes maintaining a low threshold for imaging reevaluation, involving a multidisciplinary team (surgery, hepatology, interventional radiology, gastroenterology, and infectious disease), and balancing surgical versus conservative management approaches based on individual patient risk profiles.

## Conclusions

This case highlights several key considerations in the management of cirrhotic patients presenting with potential biliary pathology. Clinicians should recognize that standard diagnostic modalities, such as abdominal ultrasound and HIDA scan, may have reduced sensitivity and specificity due to underlying hepatic dysfunction. We also recommend maintaining a low threshold for repeating imaging or using alternative imaging modalities when clinical suspicion remains high despite initial negative tests. Employing clinical judgment and collaborative decision-making to balance the immediate necessity of surgical intervention against the patient’s overall risk profile is important. Such an approach ensures that diagnoses are not missed due to confounding imaging findings and that high-risk patients receive an individualized and safely timed intervention.
